# Probability of phenotypically detectable protein damage by ENU-induced mutations in the Mutagenetix database

**DOI:** 10.1038/s41467-017-02806-4

**Published:** 2018-01-30

**Authors:** Tao Wang, Chun Hui Bu, Sara Hildebrand, Gaoxiang Jia, Owen M. Siggs, Stephen Lyon, David Pratt, Lindsay Scott, Jamie Russell, Sara Ludwig, Anne R. Murray, Eva Marie Y. Moresco, Bruce Beutler

**Affiliations:** 1 Center for the Genetics of Host Defense, University of Texas Southwestern Medical Center, Dallas, TX 75390 USA; 2 Quantitative Biomedical Research Center, Department of Clinical Science, University of Texas Southwestern Medical Center, Dallas, TX 75390 USA; 3 Kidney Cancer Program, Simmons Comprehensive Cancer Center, University of Texas Southwestern Medical Center, Dallas, TX 75390 USA; 4 Department of Statistical Science, Southern Methodist University, Dallas, TX 75205 USA; 5 Immunology Division, Garvan Institute for Medical Research, Sydney, NSW 2010 Australia

## Abstract

Computational inference of mutation effects is necessary for genetic studies in which many mutations must be considered as etiologic candidates. Programs such as PolyPhen-2 predict the relative severity of damage caused by missense mutations, but not the actual probability that a mutation will reduce/eliminate protein function. Based on genotype and phenotype data for 116,330 ENU-induced mutations in the Mutagenetix database, we calculate that putative null mutations, and PolyPhen-2-classified “probably damaging”, “possibly damaging”, or “probably benign” mutations have, respectively, 61%, 17%, 9.8%, and 4.5% probabilities of causing phenotypically detectable damage in the homozygous state. We use these probabilities in the estimation of genome saturation and the probability that individual proteins have been adequately tested for function in specific genetic screens. We estimate the proportion of essential autosomal genes in *Mus musculus* (C57BL/6J) and show that viable mutations in essential genes are more likely to induce phenotype than mutations in non-essential genes.

## Abstract (web summary)

Programs such as PolyPhen-2 predict the relative severity of damage by missense mutations. Here, Wang et al estimate probabilities that putative null or missense alleles would reduce protein function to cause detectable phenotype by analyzing data from ENU-induced mouse mutations.

## Introduction

Single-nucleotide variants (SNVs) represent an enormous source of human genetic variation, causing phenotypic differences between individuals, and less frequently, genetic disease^1–3^. On average, a human gene contains approximately four coding region SNVs, half of which are non-synonymous^4^, causing a missense mutation of the protein sequence. These single-amino-acid substitutions may alter protein function, for example, by affecting enzymatic activity, protein–protein interactions, expression, or stability, and thus lead to phenotypic changes at the level of the cell, tissue, and organism^5–8^. SNVs may also cause nonsense, makesense (stop loss), or start loss mutations resulting in deficiency of protein expression. However, understanding the contribution of individual missense mutations, as well as other types of mutations (e.g., indels), to a particular phenotype or disease is hampered by the practical challenge of testing the functional consequences of large numbers of protein variants experimentally.

To address this challenge, numerous bioinformatic tools have been developed to predict the possible impact of missense mutations on protein function^9–14^. These computational programs use direct or machine-learning strategies to variously incorporate information on protein structure, sequence, phylogeny, interaction network, and the physicochemical properties of amino acids, to infer mutational effects on protein function. Their output is a numerical and/or qualitative score that classifies mutations as benign or deleterious. Some programs combine the output of multiple prediction methods with the aim of increasing prediction accuracy^15–17^. However, a small-scale study recently found that two popular programs (PolyPhen-2 (PP2) and Combined Annotation-Dependent Depletion) misclassified as deleterious the effects of a majority of ENU-induced missense mutations in genes known to be essential for immune function, where no phenotype was detected in individual homozygous mice^18^.

To determine the probability that mutations categorized as benign or deleterious truly damage protein function, we analyze the phenotypic effects of ENU-induced mouse mutations generated as part of a large-scale mutagenesis program for forward genetic studies and cataloged in the Mutagenetix database (https://mutagenetix.utsouthwestern.edu/linksplorer/linkage_explorer.cfm). The mutagenesis pipeline provides for the genotyping, prior to phenotypic screening, of all exomic mutations present in a given pedigree, identified by whole-exome DNA sequencing of the grandsire of that lineage. A variety of phenotypic screens monitoring processes of the immune system, neurobehavior, and metabolism are applied to each mutagenized mouse.

Within a subset of known essential genes, we determine the probability that putative null alleles (indels, and nonsense, makesense, or start loss mutations caused by SNVs) and missense alleles caused by SNVs actually cause pre-weaning lethality. This allowed us to assign authentic damage probabilities to putative null alleles and to each class of missense mutations predicted by a variety of mutation effect prediction algorithms, including PP2, SIFT, LRT, MutationAssessor, FATHMM, PROVEAN, MetaSVM, MetaLR, M-CAP, and fathmm-MKL_coding, and, in turn, to directly and rationally measure genome saturation achieved through random germline mutagenesis. Overall, we find that mutation effect scores generated by prediction algorithms such as PP2 and SIFT greatly overestimate the damaging effects of missense mutations. Moreover, about 40% of putative null mutations fail to induce the expected null phenotype. We also assess the level of damage to individual genes, estimate the percentage of essential autosomal mouse genes, and show that essential genes are enriched for viable phenotypes relative to non-essential genes.

## Results

### Selection of mutations for analyses

**Fig. 1:**
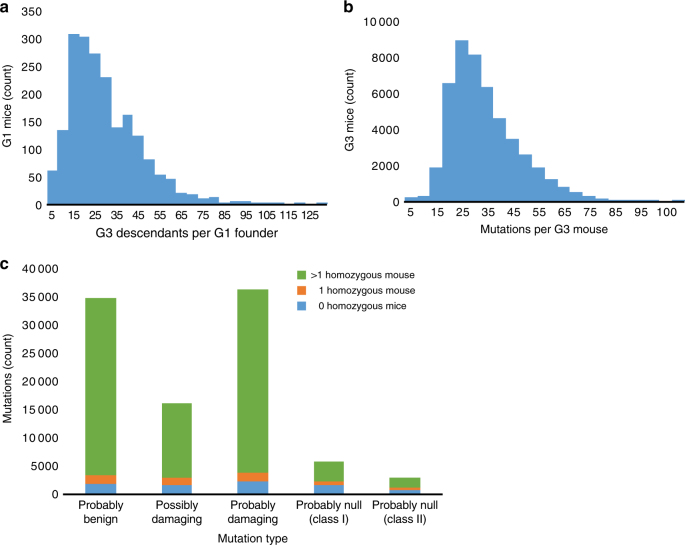
Characteristics of mice and mutations analyzed. **a** Number of G3 mice descended from the G1 male founder of each pedigree. **b** Number of heterozygous or homozygous exomic mutations in each G3 mouse. **c** Number of mutations of each mutation classification bred to homozygosity in 0, 1, or >1 mouse. Mutations were non-synonymous coding and potential splicing changes (**b**, **c**)

Our analysis utilized a selected mutation set filtered as described below from a starting population of 116,330 Ion Torrent sequencing-validated single base pair or small indel mutations from Mutagenetix, each of which occurred in at least one third-generation (G3) descendant of a mutagenized C57BL/6J male mouse. The breeding scheme to produce G3 mice carrying heterozygous and homozygous mutations (Supplementary Fig. 1) yielded, from each G1 grandsire, an average of 30 G3 offspring (Fig. 1a), each carrying about 34 mutations (Fig. 1b)^8^.

From the initial mutation set we selected 80,873 mutations that were successfully genotyped in all G2 and G3 mice of their respective pedigrees (Table 1). These mutations were located within or near (≤15 bp) coding regions and were predicted to disrupt coding and/or splicing in autosomal genes. Among them, 73,085 were missense mutations and were successfully scored by PP2 as probably damaging, possibly damaging, or probably benign (Table 1). The remaining mutations in this set (*n* = 7,788) were not scored by PP2, and we classified them as putative null alleles predicted to approximate targeted knockouts in their effects. We categorized null alleles into two classes: class I (nonsense, makesense, or start loss mutations); and class II (indels and splicing errors, both frameshift and non-frameshift) (Table 1). Eighty-two percent of the 80,873 mutations were bred to the homozygous state in at least one G3 mouse that survived to weaning age (Fig. 1c).

**Table 1:** Mutations analyzed in this study

Mutation classification	All mutations	Isolated mutations (>100 Mb from nearest neighbor)	Mutations from pedigrees with ≥3 G3 mice	Mutations in known essential genes
Probably benign (score ≤0.45)	26,004	2,406	2,311	477
Possibly damaging (score 0.45–0.95)	14,412	1,314	1,270	281
Probably damaging (score 0.95–1.0)	32,669	3,077	2,982	690
Probably null class I	5,170	462	441	78
Probably null class II	2,618	273	268	60
Total	80,873	7,532	7,272	1,586

The columns represent progressive filtering steps from left to right

We excluded closely linked mutations within a pedigree (mutations that were <100 Mb away from any other exomic mutation present on the same chromosome in the same pedigree) in order to restrict our analyses to phenotypic effects attributable to single genes. Mutations from pedigrees with <3 G3 mice were also discarded. Finally, we filtered our mutation set to keep only mutations in known essential genes (genes that when damaged in homozygous state cause pre-weaning lethality), annotated as such in the Mouse Genome Informatics (MGI) database (Supplementary Table 1). A total of 1,586 mutations in 1,027 essential genes were left for analysis after all filters had been applied (Table 1). Among them, 1,448 were missense mutations and PP2 predicted 477 to be probably benign, 281 possibly damaging, and 690 probably damaging; the remaining 138 mutations were putative null mutations, 78 of class I and 60 of class II.

### Estimating the probability of mutations being truly damaging

We assessed the genotypes of pups born to parents heterozygous for each of the 1,586 mutations and compared their frequencies to the expected Mendelian ratios to establish the damage probability for each mutation category. We used a method of moments (MM) estimator, a convenient statistical tool designed to derive parameter estimates from a sampled group of real data^19^, for estimation of the probability that mutations in each PP2 category, or putative null mutations, cause phenotypically detectable damage (pre-weaning lethality) in the homozygous state. This analysis used the HumDiv-trained PP2 algorithm (see below for comparison to HumVar-trained PP2 and other mutation prediction algorithms). The MM estimator relates the viability of homozygotes, as a measurable outcome, to protein damage, the unknown cause to be estimated. In the estimator, we classified mutations in essential genes into three types according to the frequency of lethality they are expected to cause: (1) zero homozygous mice alive at weaning, i.e., damaging mutations in totally essential genes; (2) homozygous mice alive at a percentage <25% of the total number of G3 offspring, i.e., damaging mutations in partially essential genes; and (3) homozygous mice alive at the expected Mendelian frequency of 25%, i.e., non-damaging mutations in essential genes. These assumptions should have reflected the overall distributions of truly damaging mutations and essential genes in our data, although some limitations exist; for example, other possible explanations for (2) exist, such as a gene being annotated as totally essential in a genetic background other than C57BL/6J, in which it may be only partially essential, or a particular mutation may interact with non-exonic mutations not sequenced in this project. The number of partially essential genes was estimated to be 39% of the number of totally essential genes based on the classification of 250 or 635 out of 885 MGI essential genes as causing, respectively, lethality of <100% or 100% of homozygous mutant mice (annotations of “partially essential” genes were from the International Mouse Phenotyping Consortium (IMPC) database, because they are not available in MGI annotations; Table 1). To take this into account, the MM estimator assumed the number of mutations in group (2) is 39% of the number of mutations in group (1). Applying the MM estimator with the above statistical assumptions to real data from the 1,586 mutations, we calculated the probability of protein damage to be 0.594 (0.449–0.736) for class I probably null mutations, 0.626 (0.464–0.786) for class II probably null mutations, 0.167 (0.111–0.22) for probably damaging mutations, 0.099 (0.012–0.182) for possibly damaging mutations, and 0.045 (0–0.108) for probably benign mutations (95% confidence interval (CI) all classes). Ordered from greatest to least, the estimated probabilities of protein damage corresponded in sequence to the order of mutation classifications arranged from most damaging to least damaging.

We also divided the full range of PP2 scores (0–1) into six windows and estimated damage probability using the MM estimator to determine the correlation between predicted and observed effects of mutations for narrower scoring windows than used in the initial analysis (Supplementary Table 2). For the highest and lowest scores, smaller scoring windows increased concordance between predicted and observed effects; probably benign mutations with scores 0–0.01 had 0.039 probability of protein damage, and probably damaging mutations with scores 0.999–1 had 0.199 probability of protein damage. However, narrowing the PP2 scoring windows did not dramatically alter the probabilities of protein damage calculated by the MM estimator.

**Fig. 2:**
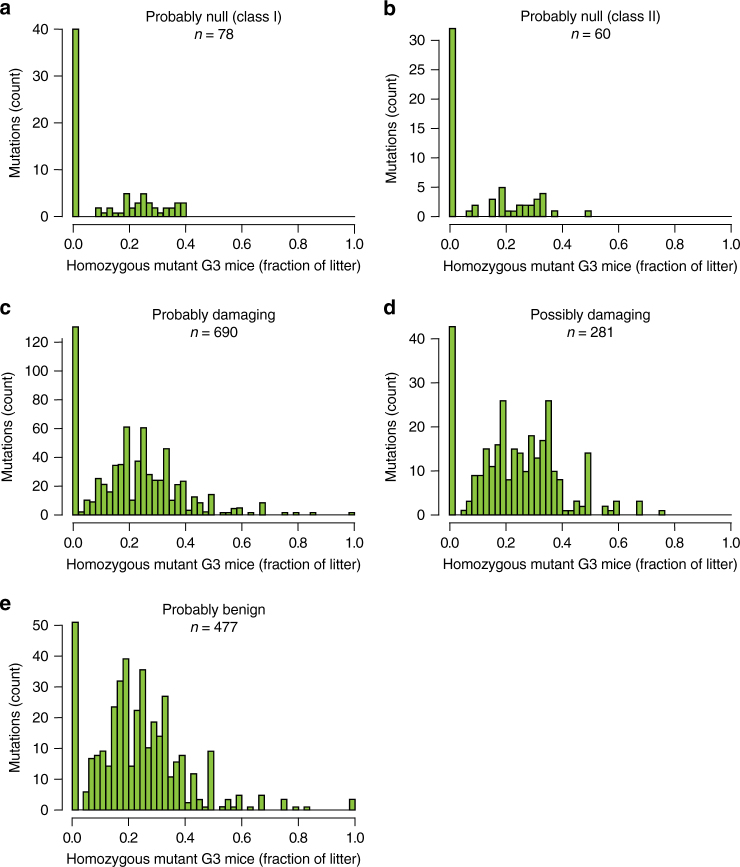
Distribution of homozygous mutant mouse frequencies among G3 mice produced by heterozygous G1 × heterozygous G2 matings. **a**–**e** For mutations of the indicated PP2 categories and putative null mutations, the proportions of homozygous mutant G3 mice resulting from heterozygous G2 matings were plotted

As an alternative approach to survey damage probability for each mutation classification, we also plotted the frequencies of homozygous mutant G3 mice per litter resulting from heterozygous G2 matings (Fig. 2), where we expected that a smaller frequency of homozygous mutant G3 mice would correspond to a higher damage probability. Consistent with this, the most damaging mutation classification (probably null) had the greatest percentage of mutations for which the frequency of viable homozygous mutant G3 mice per litter was zero (51%; Fig. 2a, b), while the least damaging classification (probably benign) had the smallest percentage of mutations for which the frequency of viable homozygous mutant G3 mice per litter was zero (10%; Fig. 2e). Strikingly, however, significant numbers of probably null mutations failed to cause 100% lethality of homozygous mutant G3 mice.

Overall, without regard to mutation class, we estimated that an autosomal ENU-induced missense or putative null mutation affecting coding/splicing causes phenotypically detectable protein damage with a probability of 16% (weighted average for all classes).

### Comparison of PP2 with SIFT and other prediction algorithms

Comparison of the effect predicted by PP2 vs. that predicted by SIFT^14^ for all missense mutations successfully scored by both PP2 and SIFT showed that of 30,337 mutations classified as deleterious by SIFT, 11.6%, 17.6%, and 70.8% were classified, respectively, as benign, possibly damaging, and probably damaging by PP2 (Supplementary Table 3). SIFT classified 57.4% of mutations as tolerated, while these were classified as benign by PP2. Thus, although PP2 and SIFT concur in classifying the damaging effects of the majority of mutations (57.4% of tolerated and 70.8% of deleterious SIFT-classified mutations had concordant PP2 classifications), PP2 and SIFT still showed a large number of discrepancies. Performing damage probability estimations using the subset of 1,586 mutations in essential genes that were successfully scored by SIFT (*n* = 1,355) resulted in probabilities of 0.085 (0.033–0.134, 95% CI) and 0.175 (0.114–0.237, 95% CI) for the “tolerated” and “deleterious” SIFT classifications, respectively (Supplementary Fig. 2).

We also evaluated other popular mutation classification algorithms, including PP2(HumVar), LRT, MutationAssessor, FATHMM, PROVEAN, MetaSVM, MetaLR, M-CAP, and fathmm-MKL_coding, using our damage probability analysis. As they are primarily designed for human variants, we lifted-over the 1,586 mutations in essential genes from mouse genome to human genome and kept for analysis only mutations that lead to the same nucleotide and amino-acid changes in both genomes. In all, 1,049 of the 1,586 mutations were successfully scored by at least one of the algorithms, using the ANNOVAR mutation annotation software^20^, and we carried out damage probability estimations for those scored by each algorithm (Supplementary Table 4). Strikingly, damage probability estimates suggested that all of the algorithms tested greatly overestimated the damaging effects of the predicted deleterious/damaging class of mutations, with the highest damage probability estimated to be 0.423 for the mutations classified “high” by MutationAssessor. In contrast, there was generally a slight underestimation of the damaging effects of predicted benign/tolerated mutations. A limitation of our analysis is the migration of the mouse mutations to the human genes and the requirement for the same nucleotide/amino-acid change, a procedural necessity for the algorithms utilized; this may result in larger damage probability estimates since it is likely that only the more “important” nucleotides and amino acids were conserved in both species. This likely explains the up to 3% difference in damage probabilities for the mouse variants vs. the humanized variants for each class of mutations classified by PP2/HumDiv. Finally, we noted that HumDiv-trained PP2 performed similarly to HumVar-trained PP2 in mutation effect prediction (Supplementary Table 4).

### Reasons putative null alleles may not cause loss of function

The surprising observation that putative null alleles of essential genes did not uniformly cause an early-lethality phenotype prompted us to examine the phenotypic effects of putative null mutations in a manually curated set of 38 non-essential genes known to cause distinctive phenotypes when mutated by a probably null mutation (Supplementary Data 1). We found that 46 (77%) of 60 putative null alleles within this group of genes elicited the expected loss-of-function phenotype in the homozygous state. We examined the nature of the mutations in the remaining 14 (23%) putative null alleles to discover possible reasons that they failed to cause the expected loss-of-function phenotypes. Of these 14 mutations, 7 were predicted to leave unaffected at least one of the protein-coding transcript isoforms encoded by the mutated gene; 2 were predicted to cause short in-frame insertions or deletions; and 2 were nonsense mutations in the final exons of the mRNA that may fail to trigger nonsense-mediated decay (Supplementary Data 1).

To systematically investigate whether such conditions affect the propensity of putative null mutations in essential genes (as annotated by MGI) to fully damage protein function, we determined whether the fraction of transcript isoforms affected by ENU-induced putative null mutations, or the position of such mutations within the linear amino-acid sequence of a protein might affect the frequency of homozygous mice observed. We found that mutations predicted to affect <30% of the total number of transcript isoforms of a gene were correlated with greater numbers of homozygous mice than mutations predicted to affect >30% of transcript isoforms (Supplementary Fig. 3a; *P* = 0.013, Student’s *t*-test). We also found that mutations near the N terminus were more likely to result in smaller proportions of viable homozygotes (Supplementary Fig. 3b; *P* = 0.0065 by a test for significance of correlation), consistent with published data^21^.

### Percentage of essential genes in the mouse genome

We wanted to estimate the fraction of essential genes in the mouse genome based on mutation-induced lethality caused by real mutations. Our overall rationale was to simulate the transmission of real mutations from real G2 mice to virtual G3 mice, varying the percentage of genes defined as essential in the simulation until the number of virtual viable G3 mice matched the observed number of viable G3 mice carrying the same mutations (Supplementary Fig. 4).

**Fig. 3:**
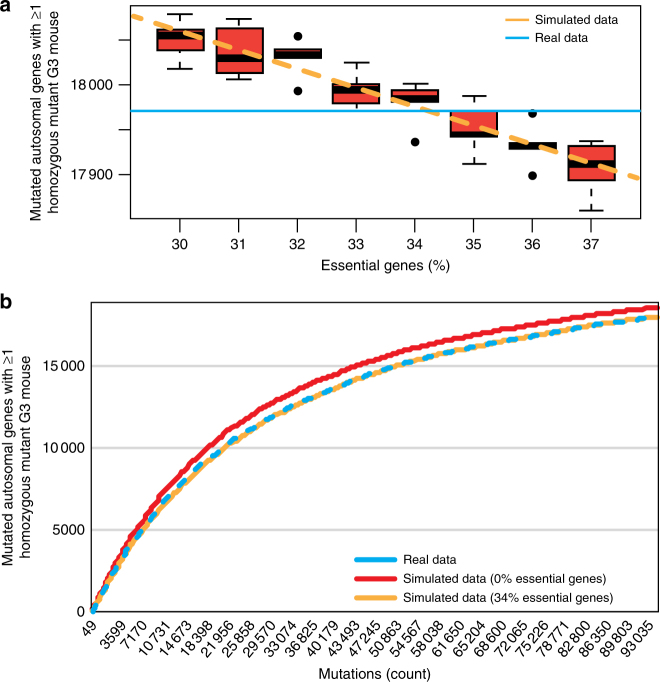
Determination of the percentage of essential genes by comparison of real and simulated lethality data. **a** Boxplot of simulated data showing the number of genes carrying any type of mutation for which at least one homozygous mutant G3 mouse existed, for varying percentages of essential genes. For each essential gene percentage, sampling was performed five times and linear regression was used to fit all the sampled data as a function of essential gene percentage. Red box represents interquartile range, and whiskers extend to the most extreme data point which is no more than 1.5 times the interquartile range; thick black line represents median. Blue line indicates the true number of genes carrying any type of mutation for which at least one HOM mouse existed. Outliers are shown as individual data points. *n* = 1,105,575 mutations analyzed. **b** Cumulative plot of the number of genes carrying any type of non-synonymous coding and potential splicing mutation for which at least one homozygous mutant G3 mouse existed vs. number of mutations using real data (blue) and simulated data, assuming essential gene percentages of 0% (red) or 34% (yellow). All five simulations were averaged for plotting curves

We used real breeding and genotype data from G0, G1, and G2 mice, and assigned litter sizes (Supplementary Fig. 5 and Materials and Methods) and genotypes to virtual G3 mice with the assumption of zero essential genes. Then, an “essential” quality was randomly assigned to a varying fraction of genes, and all mutations in such genes were designated as truly damaging or not based on their PP2 mutation category and the corresponding estimated damage probabilities. For each simulated percentage of essential genes, G3 mice were computationally “culled” if they were homozygous for designated truly damaging mutations in designated essential genes. The specified percentage of essential genes was varied until the simulation was superimposable upon the experimental data set, which occurred when the essential gene percentage was set to 34% (Fig. 3). Conducting this analysis separately for each PP2 mutation category revealed a similar essential gene percentage (30–36%) (Supplementary Fig. 6 and Supplementary Fig. 7).

### Essential genes are enriched for viable phenotypes

We speculated that lethality induced by damaging mutations of essential genes could hinder functional studies of these genes in vivo. To address this question, we analyzed mutations in essential or non-essential genes for their propensity to induce viable phenotypes. For this analysis, we used mutations of essential or non-essential genes (as annotated by MGI) that were at least 100 Mb from any other mutation on the same chromosome in the same pedigree, that were from pedigrees with at least four G3 mice of any genotype, and that were homozygous in at least 25% of G3 mice. We determined the percentage of mutations in either essential genes (*n* = 159) or non-essential genes (*n* = 882) that resulted in statistically significant phenovariance in any of 296 screens employed in the lab, as measured by linkage analysis using mutations as markers^8^ (Bonferroni-adjusted *P* values for genotype–phenotype linkage calculated using recessive, additive, or dominant transmission models; Supplementary Fig. 8). A greater percentage of mutations in essential genes than in non-essential genes was associated with at least one phenotype with a significant *P* value, despite a similar fraction of probably damaging and probably null mutations in both groups of genes (mutations in essential genes vs. mutations in non-essential genes: probably damaging, 25.8% vs. 30.5%; probably null, 5.03% vs. 5.4%). Based on the set of mutations analyzed in Supplementary Figure 8, the probability that a mutation will induce a screened phenotype (among those under surveillance in the lab^8^) with a significant linkage *P* value was 0.0527% (95% CI = 0.0477–0.0582%) for essential genes and 0.0324% (95% CI = 0.0302–0.0348%) for non-essential genes. Notwithstanding the caveat that a limited set of phenotypic screens was employed, this result suggests that mutations in essential genes have a higher chance of yielding viable phenotypes than mutations in non-essential genes.

### Genome saturation by ENU-induced protein-damaging mutations

**Fig. 4:**
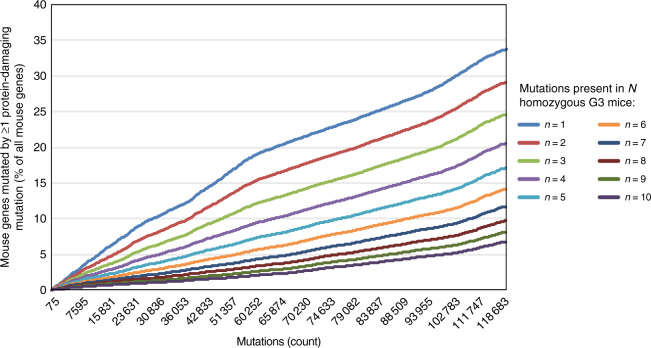
Genome saturation by 119,452 ENU-induced mutations. The estimated probability of damage for each PP2 mutation class was incorporated into the calculation of genome saturation. Cumulative plot of genome saturation percentage vs. mutation number is shown for each specified cutoff number of G3 mice carrying truly damaging homozygous mutations. Mutations were non-synonymous coding and potential splicing changes

Using programs such as PP2 or SIFT to classify mutation effects for estimation of genome saturation in forward genetic screens may result in over-estimation of the number of sufficiently damaged genes. To more accurately estimate genome saturation, we devised a methodology that incorporates the estimated probability of damage for each PP2 mutation class to calculate saturation of the mouse genome by ENU-induced protein-damaging mutations. Our approach was to assess the probability that a given gene has been damaged homozygously in one or more mice within the pedigree(s) containing mutation(s) of that gene. Then, the sum of the probabilities of damage to all mutated genes represents the expected fraction of the mouse genome damaged by the analyzed mutations. We calculated that an expected 33.7%, 29.1%, and 24.6% of all mouse genes were mutated by true protein-damaging mutation(s) in at least one, two, or three homozygous mice, respectively (Fig. 4), for the total of 119,452 recorded coding or splice site mutations in the Mutagenetix database as of 31 March 2017. This assessment of saturation can be applied to any screen, given knowledge of the mutations, their zygosity in the G3 population, and their effects as classified by PP2. As large numbers of allelic variants are created for individual genes, this method is also useful in estimating the likelihood that a particular gene has been truly functionally damaged and tested for phenotypic effect. For example, seven mutant alleles of *Ap4e1* (adaptor-related protein complex AP-4, epsilon 1) in our collection have been bred to homozygosity in 32 G3 mice; based on the probability of damage by these mutations, we calculated about 71% probability that *Ap4e1* was functionally damaged or destroyed and examined in three or more mice (Supplementary Fig. 9).

## Discussion

We found that mutation effect scores generated by prediction algorithms such as PP2 and SIFT, which have not previously been translated to absolute estimates of likelihood of damage, greatly overestimated the damaging effects of missense mutations. In support of this, based on comparison of the predicted and actual effects of 2,314 TP53 missense mutations on transactivation activity, one study found that a substantial fraction of mutations classified as deleterious by various prediction programs showed only subtle effects on TP53 transactivation activity^18^. For mutations in essential genes, it may be that the qualitative phenotypic measure we utilized (lethality and sub-viability) precluded observation of subtle effects, thereby magnifying the apparent discrepancy between predicted and observed effects of mutations. Tissue- or system-specific phenotypic screens may disclose more fully the damaging nature of predicted deleterious mutations. False-positive predictions of deleterious effects may also reflect permissive positions within the protein sequence, for which substitutions may not or may minimally affect protein function(s) despite being evolutionarily conserved or meeting other PP2/SIFT criteria as important.

Somewhat surprisingly, putative null mutations of essential genes induced lethality of all homozygous mice within a given pedigree an average of only 61% of the time. This observation was supported by the finding that 23% of putative null alleles within a small, curated set of non-essential genes failed to produce expected phenotypic effects although targeted knockout alleles of the same genes were known to do so. This suggests that putative null alleles induced by ENU may be less robust in their destructive effects than targeted knockout alleles. We found evidence that putative null alleles may not fully destroy the encoded protein when they occur toward the C terminus, possibly because truncations close to the end of the coding sequence preserve some protein function. We found that putative null alleles affecting 30% or fewer of the transcript isoforms of a gene also may not fully destroy protein function. In addition, it is possible that mutations affecting critical splice junction nucleotides may permit some normal splicing to occur, along with alternative splicing that yields a functional protein product; premature stop codons and indels that alter reading frame may be phenotypically suppressed by alternative splicing; and makesense alleles, while usually destabilizing, may also occasionally yield functional protein products.

Our conclusions concerning the damaging effects of mouse mutations presumably also apply to human mutations and emphasize the importance of experimental validation of mutation effects in the assessment of the etiology of germline genetic diseases or cancer. On a practical level, an informed decision based on actual probabilities of protein damage caused by mutations of the various classes is necessary to set cutoff scores for filtering mutations to be tested experimentally. Supplementary Table 4 documents the estimated damage probabilities of each class of mutations predicted by a variety of algorithms, including PP2, SIFT, LRT, MutationAssessor, FATHMM, PROVEAN, MetaSVM, MetaLR, M-CAP, and fathmm-MKL_coding, and could serve as a useful resource for this purpose. A limitation of our study is the assumption that reduced frequencies of viable homozygous mutant G3 mice in a litter (expected frequencies <25% but >0%) are only due to mutations of partially essential genes. Other possible explanations exist; for example, a gene may be annotated as totally essential in a genetic background other than C57BL/6J, in which it may be only partially essential; or, a particular mutation may interact with non-exonic mutations not sequenced in this project. Given limited data, it is impossible to consider all scenarios in our estimations. However, the correspondence between the magnitude of calculated damage probabilities and the severity of predicted effects of mutations, and the concordance between our estimates and previously published essential mouse gene percentages, suggests that these issues have not dominantly biased our damage probability estimates.

To estimate the proportion of essential genes in the mouse genome, we took a novel approach in which we simulated the transmission of actual mutations and the survival of virtual G3 mice to which they were transmitted, varying the essential gene percentage applied in the simulation until numbers of homozygously damaged proteins matched observed numbers. Our estimate of the proportion of essential genes (34%) was similar to estimates based on gene targeting^22–24^ and other methods^25^, supporting the validity of our estimates of damage probability and the overall rationale of the simulation. However, in contrast to determinations of essential gene percentage based on lethality of knockout mouse lines^22–24, 26^, our approach may be considered unbiased in that it required no gene selection, a process that may skew the range and severity of phenotypes observed. Moreover, due to the random nature of ENU mutagenesis across the whole genome, our approach avoids inaccuracy that may be introduced by the reported region-to-region and chromosome-to-chromosome variation in the density of essential mouse genes^25^. Importantly, the essentiality simulation validates our approach to estimation of genome saturation, both of which were based on a similar paradigm of incorporating damage probability estimates, which can be widely applied in other forward genetic screening projects.

Although essential genes might be considered genomic “dark matter”, invisible to forward genetic analysis because homozygous mice carrying truly damaging mutations would be expected to show a lethal phenotype, we found that essential genes were enriched for viable recessive phenotypes compared to non-essential genes. This is especially striking given that our analysis was limited to mutations that were transmitted to G3 mice at a minimum frequency of 25% (expected Mendelian frequency), which means that a large fraction of truly damaging mutations in partially essential genes were excluded from the comparison. In mice, homozygous mutations in partially essential genes have been reported to yield more phenotypes than homozygous mutations in non-essential genes^22^. Moreover, several studies reported that essential mouse genes were enriched among disease genes when their human orthologs were examined for disease associations^22, 27, 28^. Essential genes, required for survival at one or more stages of pre-weaning development, may be more pleiotropic than non-essential genes, the consequences of which are relatively localized or focused phenotypes for hypomorphic alleles and lethality for null alleles.

## Methods

### Breeding and genotyping of ENU-mutagenized mice

Five- to six-week old C57BL/6J mice were purchased from The Jackson Laboratories. ENU mutagenesis was performed by injecting male mice intraperitoneally with 100 mg ENU/kg of body weight once per week for 3 weeks. After the last injection, the mice were housed one per cage for 12 weeks to allow for recovery of fertility^29^. Mutagenized G0 males were bred to C57BL/6J females or to female mice that carried ENU-induced mutations from their G0 fathers (termed G0′; Supplementary Fig. 1). The resulting G1 males were crossed to C57BL/6J females to produce G2 mice. G2 females were backcrossed to their G1 sires to yield G3 mice. The viability of G3 mice was determined at weaning (28 days of age). In the G3 generation, with regard to individual ENU-induced mutations, homozygotes for the reference allele, heterozygotes, and homozygotes for the variant allele are referred to as REF, HET, and HOM mice, respectively. Mice were maintained within the Animal Resource Center of the University of Texas Southwestern Medical Center. All experimental procedures using mice were approved by the Institutional Animal Care and Use Committee of the University of Texas Southwestern Medical Center and were conducted in accordance with the institutionally approved protocols and guidelines for animal care and use.

Every G1 mouse was subjected to whole-exome sequencing as previously described^8^. A total of 58 755 622 bp were targeted for whole-exome sequencing using oligonucleotide probes from Life Technologies’ TargetSeq Custom Enrichment Kit and modified to run on an Illumina HiSeq 2500 platform. Paired-end 2 × 100-bp sequencing was performed using an Illumina HiSeq 2500 instrument to detect heterozygous autosomal and hemizygous X-linked mutations. Reads were demultiplexed using CASAVA according to their index sequence and lane numbers. Reads were mapped to the University of California Santa Cruz mm10 genome reference sequence for C57BL/6J using Burrows-Wheeler Aligner v0.7.10. Duplicate reads were removed by SAMtools and indel regions left aligned by Genome Analysis Toolkit. Coverage was calculated over targeted regions using BEDTools. Variants relative to the C57BL/6J reference sequence (GRCm38) were called and annotated by a combination of SAMtools, SnpEff, SnpSift, and ANNOVAR, and then filtered to eliminate SNPs listed in dbSNP (build 137) and common variants observed in a rolling total of 40 previously sequenced mice with unshared G0 sires. Synonymous mutations and mutations not predicted to affect splicing or coding sense were also eliminated. Remaining mutations with a quality score ≥20 were listed in BED format and targeted in AmpliSeq panel design. On average, coverage of the composite coding region was such that 97% of all coding nucleotides were sequenced 10× or more times. The G2 females and all G3 mice were then genotyped at all mutation sites predicted to change the protein-coding sequence (i.e., missense, nonsense, makesense, start loss, splicing errors, and small indels) that showed a quality score exceeding 20. The rate of validation for all mutations tested was 93.9%. For mutations with a quality score >80 (encompassing 91.96% of all mutations), the rate of validation was 99.3%. While no attempt was made to validate synonymous mutations, >99% of these mutations were presumed authentic if their quality score exceeded 80. Mutations were validated and genotypes were determined in G1, G0′ (when available), G2, and G3 mice using AmpliSeq custom panels and Ion Torrent sequencing. All loci were amplified in single PCR via custom AmpliSeq panel primer mixes. The amplicons were made into Ion Torrent barcoded libraries and run on the Ion PGM (Life Technologies) in 316 or 318 chips via 200-bp sequencing. Alignment was performed by TMAP software within the Torrent Suite Software package to the UCSC mm10 genome reference sequence for C57BL/6J. Variants were called using the Torrent Variant Caller plugin available in Torrent Suite software, and an output file was generated containing the total number of reads for REF and VAR alleles for each barcoded sample. All putative mutations that were shown to be false positives (i.e., genotyped as homozygous reference in both the G1 and a wild-type control sample) were eliminated from further consideration. For estimating probability of damage, only autosomal genes were analyzed because the X chromosome was not mutagenized in G1 mice that did not have a G0′ dam.

### Classification of mutation types

All missense alleles were evaluated and scored by PP2 (HumDiv-trained)^30^, which was modified for evaluation of mouse mutations according to the README file accompanying the program download. Briefly, PP2 was provided with mouse entries from the Uniprot and Pfam databases, then multiple sequence alignments were built using the mouse sequences. Non-missense mutations predicted to affect coding or splicing were not scored by PP2, and were classified as probably null and divided into two categories: class I (nonsense, makesense, or start loss mutations); and class II (splicing errors and indels (up to 12 bp in length) predicted to cause coding or splicing errors, both frameshift and non-frameshift). Effects of mutations up to 100 bp from an exon boundary on splicing of transcripts were evaluated by a splice site prediction program based on the maximum entropy model developed by Yeo and Burge^31^, in which scores are assigned to 9-mer splice donor sites and 23-mer splice acceptor sites. Higher scoring sequences have a greater probability of being used in splicing. All ENU-induced mutations were evaluated for their effect on the score of native sequences. Mutations predicted to disrupt native splice sites leading to exon skipping or use of a cryptic splice site were classified as probably null. Intronic mutations not predicted to affect splicing were classified as probably benign.

### Identifying a list of previously known essential genes

We retrieved viability annotations from MGI and IMPC databases. Specifically, a batch query requested all mouse genes with Mammalian Phenotype (MP) information from MGI. A gene was classified as essential if at least one of the MP annotations reported lethality associated with an induced mutation of any kind, yielding a list of 3555 essential genes (Supplementary Table 1; retrieved August 2016; http://www.informatics.jax.org/batch)^32^. Viability annotations for 2729 genes from the IMPC database were also retrieved (retrieved August 2016; http://www.mousephenotype.org/data/batchQuery)^33^; some genes had conflicting records within the IMPC annotations and were discarded from further analysis. The remaining 2691 genes were classified as viable (1784; 66.3%), sub-viable (258; 9.6%), or lethal (649; 24.2%). We compared annotations for the set of genes annotated in both databases and found that MGI annotations were on average 95.5% concordant with those of IMPC (Supplementary Table 5). Because of its larger size, we used the essential genes list from MGI to filter our mutation set, resulting in 1586 mutations in 1027 MGI essential genes.

Among the genes classified as essential by MGI, IMPC classified 635 as totally lethal and 250 as partially lethal when damaged. Therefore, we assumed the proportion of partially essential genes to be 39% (250/635) of totally essential genes.

### Calculation of damage probability for each mutation class

ENU-induced mutations in essential genes with complete genotypic data for both G2 and G3 mice were filtered to remove those mutations <100 Mb away from any other mutation on the same chromosome within the same pedigree, which left mutations that were meiotically unlinked from all other identified coding/splicing mutations in the same pedigree (Table 1). The mutations were further filtered to exclude those from pedigrees with fewer than three G3 mice of any genotype. Finally, for each damage category (probably damaging, possibly damaging, probably benign, and probably null), the proportion (*p*_i_) of G3 HOM mice out of all G3 mice in the same litter(s) was calculated for each mutation.

Three subgroups of mutations were assumed to exist:

1. One subgroup of proportion (*ρ*) contained mutations in which the proportion of HOM mice is 0. These represent mutations in essential genes that induce a totally lethal effect.
2. One subgroup of proportion ($0.39\rho$) contained mutations in which the proportion of HOM mice are randomly distributed around *θ*. These represent mutations in essential genes that induce partial lethal effect. *θ* is a variable between 0 and 0.25. The true value for it is unknown so we used *θ* = 0.125 for this study.
3. One subgroup ($1 - 0.39\rho$) contained mutations that do not damage the proteins and the proportion of HOM mice is distributed around 0.25.

The probability of each class of mutations being truly damaging was calculated by a MM estimator ($\rho _{{\rm MM}} = 1.39 \times \frac{{\frac{1}{4} - \frac{{{\sum} {p_{\rm i}} }}{n}}}{{\frac{1}{4} \times 1.39 - \frac{1}{8} \times 0.39}}$), where *p*_i_ refers to the proportion of HOM mice for each mutation and *n* refers to the total number of mutations in each category. The confidence interval was derived from 5000 bootstrap samplings.

### Simulation of ENU mutagenesis

Simulation of ENU mutagenesis was accomplished by using breeding and genotype data from the G0, G1, and G2 mice available in the Mutagenetix database (retrieved March 2017; http://mutagenetix.utsouthwestern.edu); G3 mice were “bred” in silico by a random process. To determine the number of G3 mice in each pedigree when all genes were hypothetically neutral, we fit a linear regression model of pedigree sizes regressed by pedigree type, number of litters produced, and number of each type of mutations in each G2 mother (Supplementary Fig. 5). Then, all mutation counts were set to zero, which is equal to the assumption that all mutations in the genes were neutral. Next, the simulation randomly generated G3 mice and their genotypes from in silico “mating” based on the genotypes of the G2 mother and father, and the hypothetical pedigree sizes. Then, an “essential” quality was randomly assigned to a varying fraction of genes, and all mutations in such genes were designated as truly damaging or not based on their PP2 mutation category and the corresponding estimated damage probabilities. The assigned essential genes were further sampled to be “partially essential” or “totally essential” with a ratio of 39:100. For each simulated percentage of essential genes, G3 mice were computationally “culled” if they were homozygous for designated truly damaging mutations in designated totally essential genes; G3 mice homozygous for designated truly damaging mutations in designated partially essential genes were culled at a rate of 50%.

### Calculation of genome saturation by damaging mutations

We employed a straightforward method to calculate the saturation of the genomic target, building upon the probability of damaging a certain gene *g* in the homozygous state in at least *n* G3 mice (*n* = 1, 2,…,) from one or multiple pedigrees. When analyzing mutation data from a single pedigree or a superpedigree (combined pedigrees containing a common mutated gene) for a certain gene *g*, we denote all mutations to be analyzed as *mut*_*j*_ (*j* = 1…*J*) and all G3 mice to be analyzed as *mouse*_*k*_ (*k* = 1…*K*) . We use a matrix, *M*, of *J* × *K* dimensions to denote the mutation status of these mice in the mutations. *M*_*jk*_ = 1 is mouse *k* is homozygous for mutation *j*; otherwise,*M*_*jk*_ = 0. Note the same mutation can be shared across different mice and even different pedigrees and the same mouse can have more than one mutation in the target gene.

When *J* ≤ 10, we go through every enumeration where each unique mutation *m*_*j*_ was set to be truly damaging or not with probability *P*(*m*_*j*_) . Then given the assumed damaging status of all mutations, we calculate the number of mice,$c_{m_1,m_2,m_3 \ldots m_J}$, carrying at least one truly damaging homozygous mutation of the gene in question. Then the probability of damaging at least *n* mice in one pedigree or one superpedigree is

$$P_g(n) = \mathop {\sum}\limits_{m_{1 = 0,1}} {\mathop {\sum}\limits_{m_{2 = 0,1}} {\mathop {\sum}\limits_{m_{3 = 0,1}} {...\mathop {\sum}\limits_{m_{J = 0,1}} {P(m_1)P(m_2)P(m_3)...P(m_J)I\left( {c_{m_1,m_2,m_3...m_J} \ge n} \right)} } } }$$

When $J > 10$, we will carry out 1,000 Monte Carlo simulations. In each simulation, each unique mutation *m*_*j*_ was sampled to be truly damaging or not depending on their mutation category and previously calculated damaging probability. Then given the sampled damaging status of all mutations, we calculate the number of mice carrying at least one truly damaging homozygous mutation of the gene in question. Then we will obtain a vector of integer numbers *C*=[*c*_1_,*c*_2_,…,*c*_1000_], each element of which is the number of homozygously mutated mice. The probability of damaging more than *n* mice in one pedigree or one superpedigree is estimated to $P_g(n) = \frac{{\mathop {\sum}\limits_{s = 1...1000} {I\left( {c_s > = n} \right)} }}{{1000}}$.

Building on $P_g(n)$, the expected number of all mouse genes damaged within the entire mutagenesis effort in at least *n* HOM mice is $S = \mathop {\sum}\nolimits_{g = 1 \ldots G} {P_g(n)}$. The saturation can be calculated at each time point in the whole process of the screening project and plotted in a cumulative manner. The saturation calculation and plotting functionalities have been implemented on the Mutagenetix website (Supplementary Fig. 9, https://mutagenetix.utsouthwestern.edu/report/gene_damage/damage_prob.cfm).

### Calculation of the probability of inducing viable phenotypes

We investigated all phenotypic screens conducted for the same set of mutations as analyzed in Supplementary Figure 8 in essential genes and non-essential genes. We calculated the percentage of screen/mutation combinations with linkage *P* values <1 × 10^−5^ over all available screen/mutation combinations. We used these percentages as the estimates of probability of inducing putative phenotype for mutations in essential genes and non-essential genes in our mutagenesis screening project. All recessive, additive, and dominant phenotypes were considered. Confidence interval was calculated from binomial distribution.

### Code availability

Core analysis codes are deposited in GitHub: https://github.com/wtwt5237/Probability-of-phenotypically-detectable-protein-damage-.git.

### Data availability

Summary-level mutation, genotype, and phenotype data are available in the Mutagenetix database (https://mutagenetix.utsouthwestern.edu/). Comprehensive detailed data are available upon request from the authors.

## Electronic supplementary material

Supplementary Information

Description of Additional Supplementary Files

Supplementary Data 1

## References

[1] Lek M (2016). Analysis of protein-coding genetic variation in 60706 humans. Nature.

[2] 1000 Genomes Project Consortium (2015). A global reference for human genetic variation. Nature.

[3] 1000 Genomes Project Consortium (2010). A map of human genome variation from population-scale sequencing. Nature.

[4] Cargill M (1999). Characterization of single-nucleotide polymorphisms in coding regions of human genes. Nat. Genet..

[5] Turer E (2017). Creatine maintains intestinal homeostasis and protects against colitis. Proc. Natl Acad. Sci. USA.

[6] Zhang Z (2016). Insulin resistance and diabetes caused by genetic or diet-induced KBTBD2 deficiency in mice. Proc. Natl Acad. Sci. USA.

[7] Shi H (2016). NLRP3 activation and mitosis are mutually exclusive events coordinated by NEK7, a new inflammasome component. Nat. Immunol..

[8] Wang T (2015). Real-time resolution of point mutations that cause phenovariance in mice. Proc. Natl Acad. Sci. USA.

[9] Zhao N, Han JG, Shyu CR, Korkin D (2014). Determining effects of non-synonymous SNPs on protein-protein interactions using supervised and semi-supervised learning. PLoS Comput. Biol..

[10] Adzhubei, I., Jordan, D. M. & Sunyaev, S. R. Predicting functional effect of human missense mutations using PolyPhen-2. *Curr. Protoc. Hum. Genet*. Chapter **7**, Unit 7.20 (2013). 10.1002/0471142905.hg0720s76 PMC4480630 23315928

[11] Gnad F, Baucom A, Mukhyala K, Manning G, Zhang Z (2013). Assessment of computational methods for predicting the effects of missense mutations in human cancers. BMC Genomics.

[12] Khurana E, Fu Y, Chen J, Gerstein M (2013). Interpretation of genomic variants using a unified biological network approach. PLoS Comput. Biol..

[13] Reva B, Antipin Y, Sander C (2011). Predicting the functional impact of protein mutations: application to cancer genomics. Nucleic Acids Res..

[14] Kumar P, Henikoff S, Ng PC (2009). Predicting the effects of coding non-synonymous variants on protein function using the SIFT algorithm. Nat. Protoc..

[15] Lopes MC (2012). A combined functional annotation score for non-synonymous variants. Hum. Hered..

[16] Gonzalez-Perez A, Lopez-Bigas N (2011). Improving the assessment of the outcome of nonsynonymous SNVs with a consensus deleteriousness score, Condel. Am. J. Hum. Genet..

[17] Li MX (2013). Predicting mendelian disease-causing non-synonymous single nucleotide variants in exome sequencing studies. PLoS Genet..

[18] Miosge LA (2015). Comparison of predicted and actual consequences of missense mutations. Proc. Natl Acad. Sci. USA.

[19] Bowman, K. O. & Shenton, L. R. in *Encyclopedia of Statistical Sciences *(eds Kotz, S., Read, C. B., Balakrishnan, N. & Vidakovic, B.) 2092–2098 (Wiley, 1998).

[20] Wang K, Li M, Hakonarson H (2010). ANNOVAR: functional annotation of genetic variants from high-throughput sequencing data. Nucleic Acids Res..

[21] MacArthur DG (2012). A systematic survey of loss-of-function variants in human protein-coding genes. Science.

[22] Dickinson ME (2016). High-throughput discovery of novel developmental phenotypes. Nature.

[23] Ayadi A (2012). Mouse large-scale phenotyping initiatives: overview of the European Mouse Disease Clinic (EUMODIC) and of the Wellcome Trust Sanger Institute Mouse Genetics Project. Mamm. Genome.

[24] White JK (2013). Genome-wide generation and systematic phenotyping of knockout mice reveals new roles for many genes. Cell.

[25] Hentges KE, Pollock DD, Liu B, Justice MJ (2007). Regional variation in the density of essential genes in mice. PLoS Genet..

[26] Hrabe de Angelis M (2015). Analysis of mammalian gene function through broad-based phenotypic screens across a consortium of mouse clinics. Nat. Genet..

[27] Georgi B, Voight BF, Bucan M (2013). From mouse to human: evolutionary genomics analysis of human orthologs of essential genes. PLoS Genet..

[28] Dickerson JE, Zhu A, Robertson DL, Hentges KE (2011). Defining the role of essential genes in human disease. PLoS ONE.

[29] Georgel P, Du X, Hoebe K, Beutler B (2008). ENU mutagenesis in mice. Methods Mol. Biol..

[30] Adzhubei IA (2010). A method and server for predicting damaging missense mutations. Nat. Methods.

[31] Yeo G, Burge CB (2004). Maximum entropy modeling of short sequence motifs with applications to RNA splicing signals. J. Comput. Biol..

[32] Blake JA (2003). MGD: the Mouse Genome Database. Nucleic Acids Res..

[33] Brown SD, Moore MW (2012). The International Mouse Phenotyping Consortium: past and future perspectives on mouse phenotyping. Mamm. Genome.

