# Supplementary material for: Probability of phenotypically detectable protein damage by ENU-induced mutations in the Mutagenetix database
Source: Nat Commun. 2018 Jan 30;9:441. doi: 10.1038/s41467-017-02806-4 (PMC5789985; doi:10.1038/s41467-017-02806-4)
Supplement: Supplementary file 1 — Supplementary Information [file 41467_2017_2806_MOESM1_ESM.pdf]

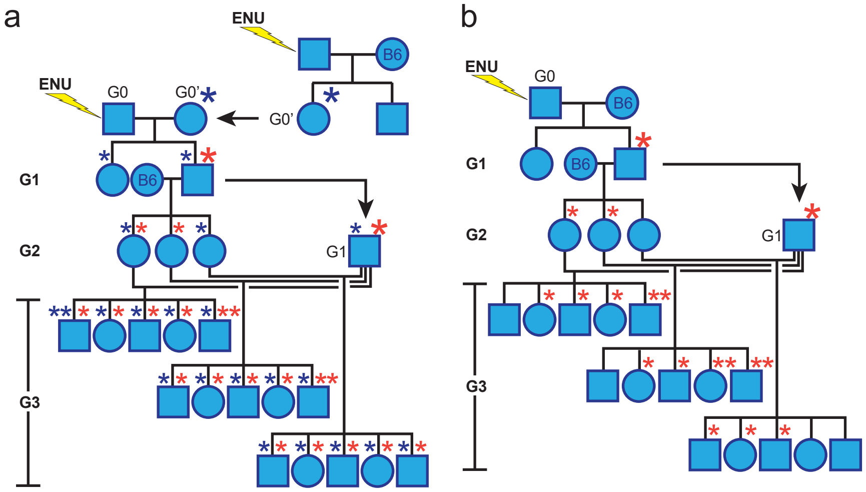

Supplementary Figure 1. Breeding scheme to produce G3 mice carrying heterozygous and homozygous ENU induced mutations. Mutagenized G0 males were bred to (a) G0' females carrying mutations derived from other mutagenized males, or (b) C57BL/6J (B6) females. The resulting G1 males were crossed to B6 females to produce G2 mice. G2 females were backcrossed to their G1 sires to yield G3 mice. Every G1 male founder was subjected to whole exome sequencing and its G2 and G3 descendants were genotyped at the identified mutation sites. Asterisks indicate mutations originating from the G0 male (red) or G0' female (blue), with larger asterisks representing initial germline transmission.

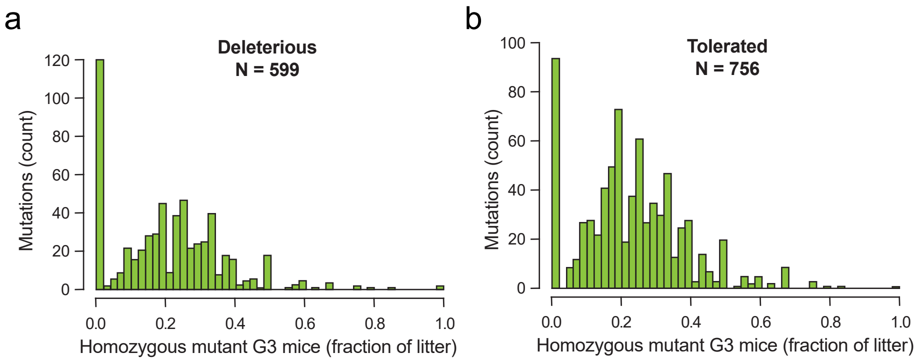

Supplementary Figure 2. Distribution of homozygous mutant mouse frequencies among G3 mice produced by heterozygous G2 matings for SIFT-classified mutations. (a-b) For mutations classified (a) deleterious or (b) tolerated by SIFT, the proportions of homozygous mutant G3 mice resulting from heterozygous G2 matings were plotted.

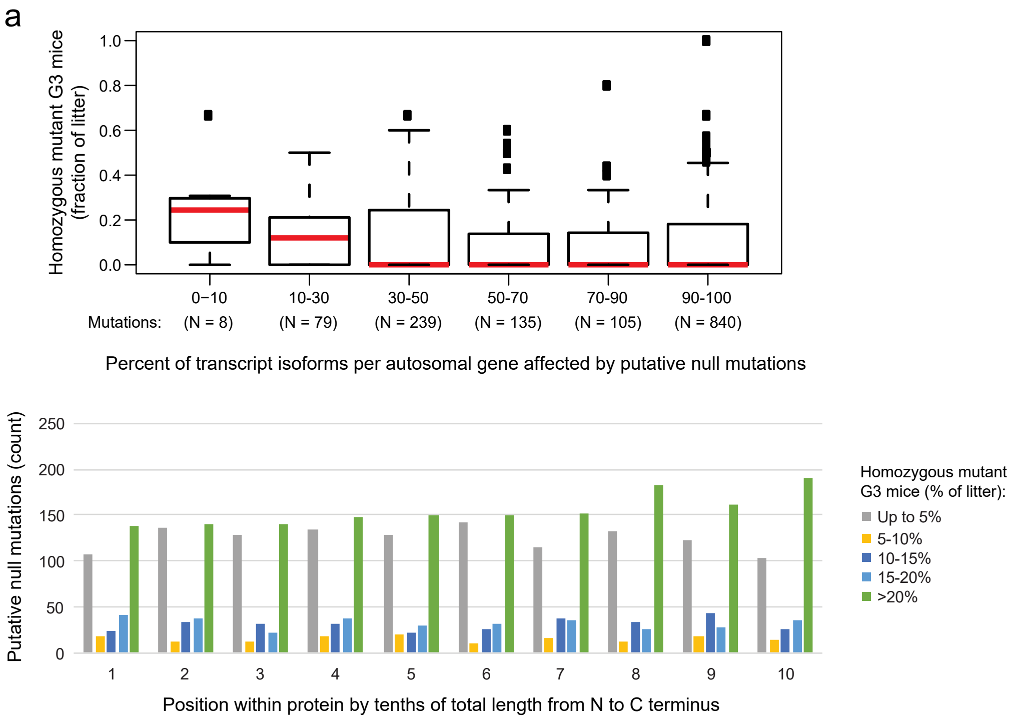

Supplementary Figure 3. Effect of position of putative null mutations within the linear amino acid sequence of a protein or fraction of transcript isoforms affected on the propensity to cause functional damage. (a-b) Essential genes were analyzed. (a) The proportions of homozygous mutant G3 mice resulting from heterozygous G2 matings plotted versus the percentage of transcript isoforms of a gene affected by putative null mutations. Boxplots generated as in Fig. 3. (b) The number of mutations positioned within each protein segment (by tenths of total amino acid length) and that were present in homozygous state in ≤5%, 5-10%, 10-15%, 15-20%, or >20% of G3 mice produced by heterozygous G2 matings. The position within the protein was obtained by dividing the number of amino acids N-terminal to the mutation by the full length of the non-mutated protein.

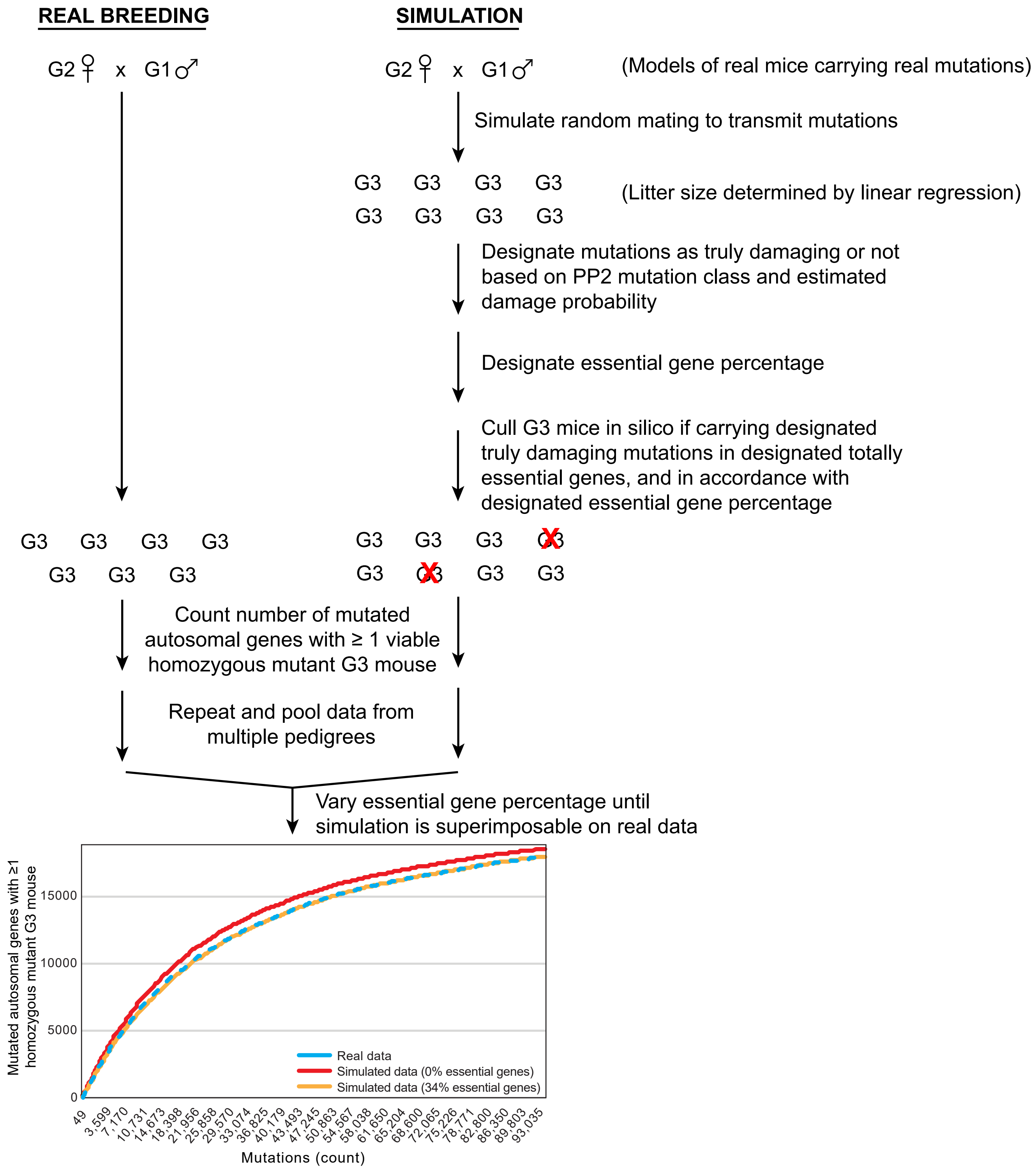

Supplementary Figure 4. Schematic of simulation to determine the percentage of essential genes in the mouse genome.

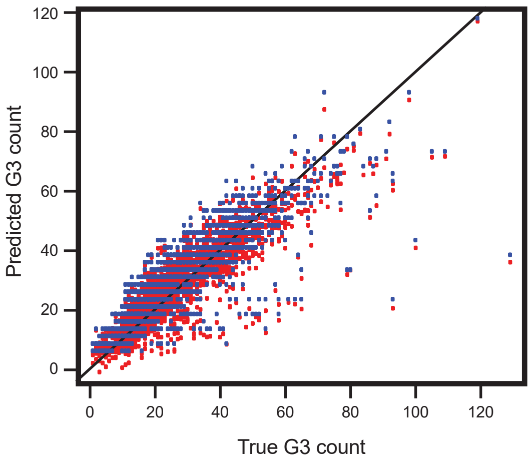

Supplementary Figure 5. Predicted pedigree sizes assuming no genes are essential. Pedigree size refers to the number of G3 mice in a litter. Linear regression model of true pedigree size (red) regressed by pedigree type, number of litters produced, and number of each type of mutations. A total of 2,005 pedigrees were used in the prediction. All mutations counts were then assumed to be zero, which is equal to assuming no mouse genes are essential, and the regression model predicted the hypothetical pedigree sizes (blue).

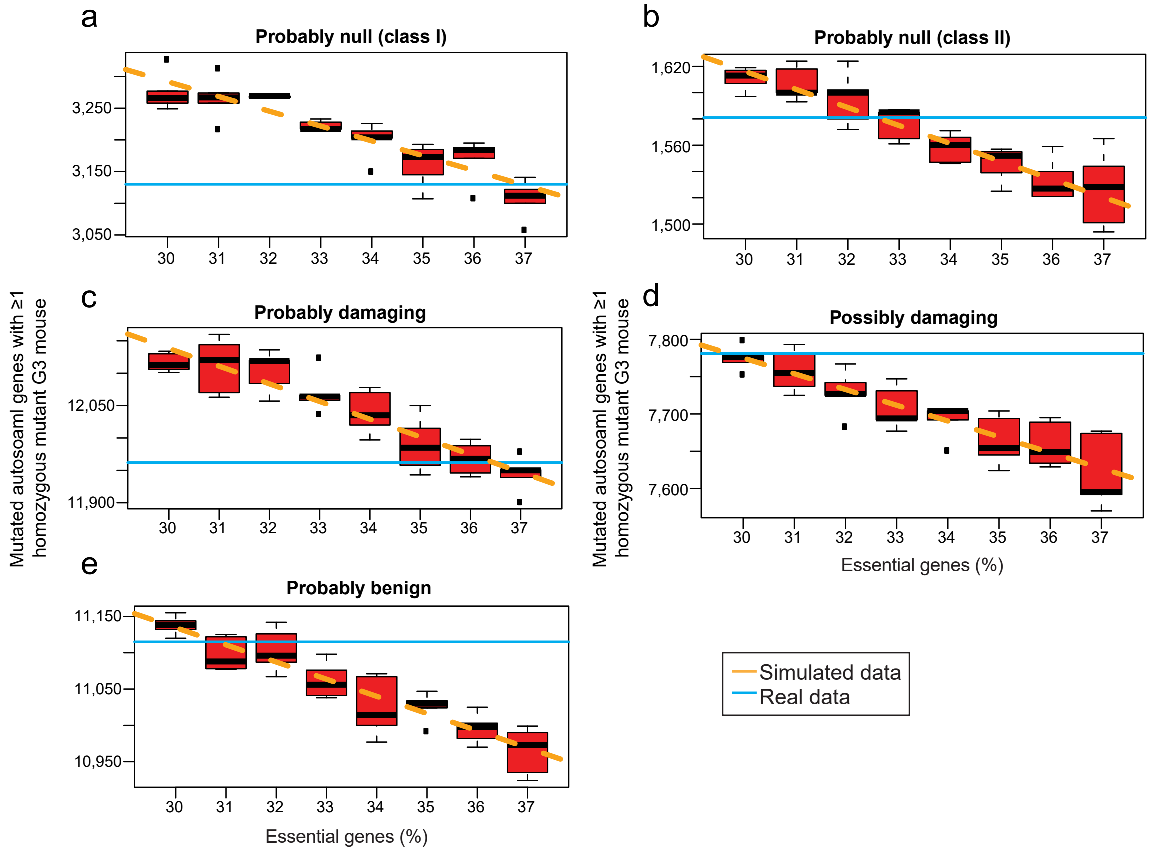

Supplementary Figure 6. Determination of the percentage of essential genes by comparison of real and simulated lethality data. Simulated data are plotted showing the number of genes carrying (a) probably null class I, (b) probably null class II, (c) probably damaging, (d) possibly damaging, or (e) probably benign mutations for which at least one homozygous mutant G3 mouse existed, for varying percentages of essential genes. For each essential gene percentage, sampling was performed five times and linear regression was used to fit all the sampled data as a function of essential gene percentage. Boxplots generated as in Fig. 3. Blue line indicates the true number of genes carrying mutations of the indicated classification for which at least one HOM mouse existed. N = 1,105,575 mutations analyzed.

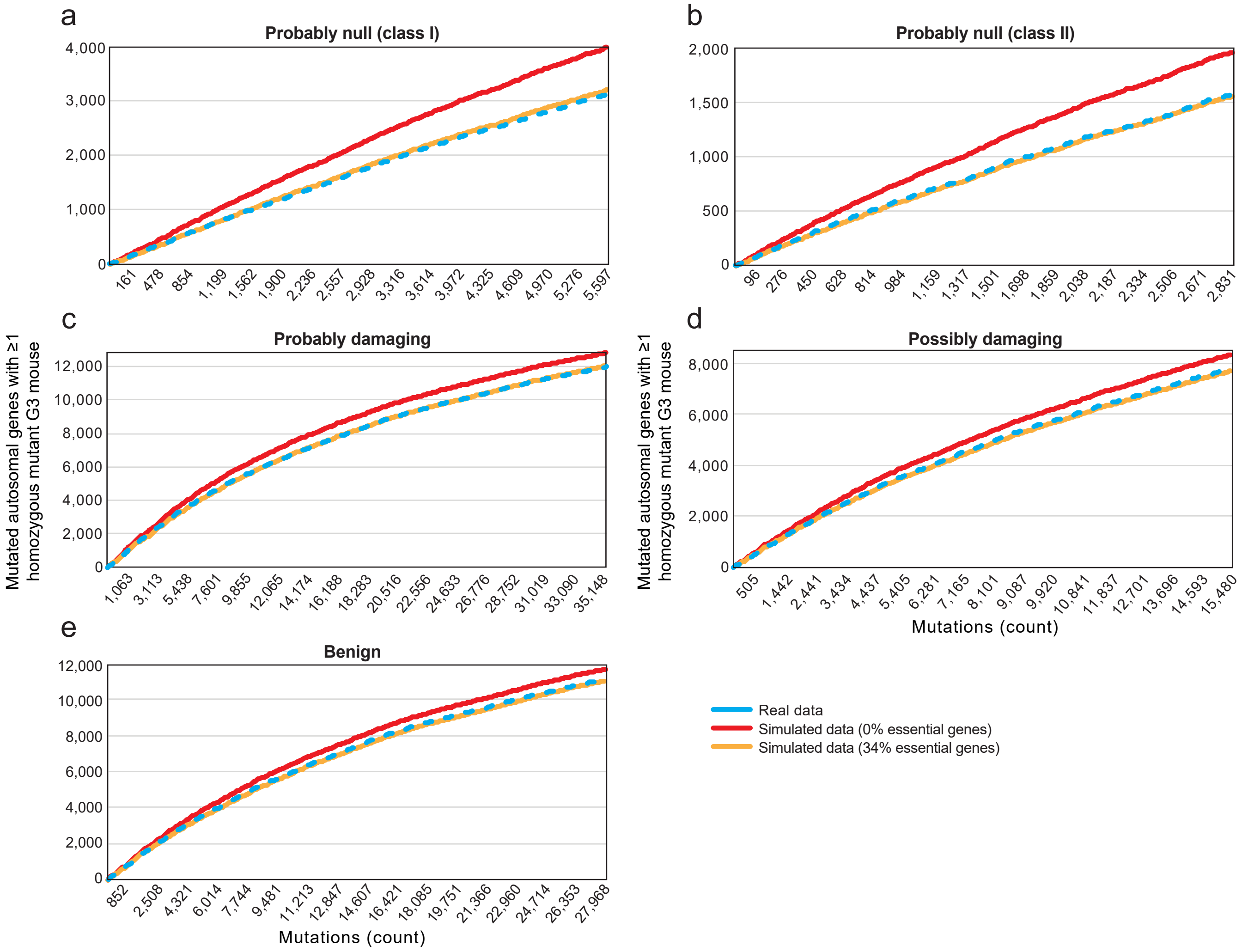

Supplementary Figure 7. Determination of the percentage of essential genes by comparison of real and simulated lethality data. Cumulative plot of the number of genes carrying (a) probably null class I, (b) probably null class II, (c) probably damaging, (d) possibly damaging, or (e) probably benign mutations for which at least one homozygous mutant G3 mouse existed versus number of mutations using real data (blue) and simulated data, assuming essential gene percentages of 0% (red) or 34% (yellow). Each curve is the average of five simulations.

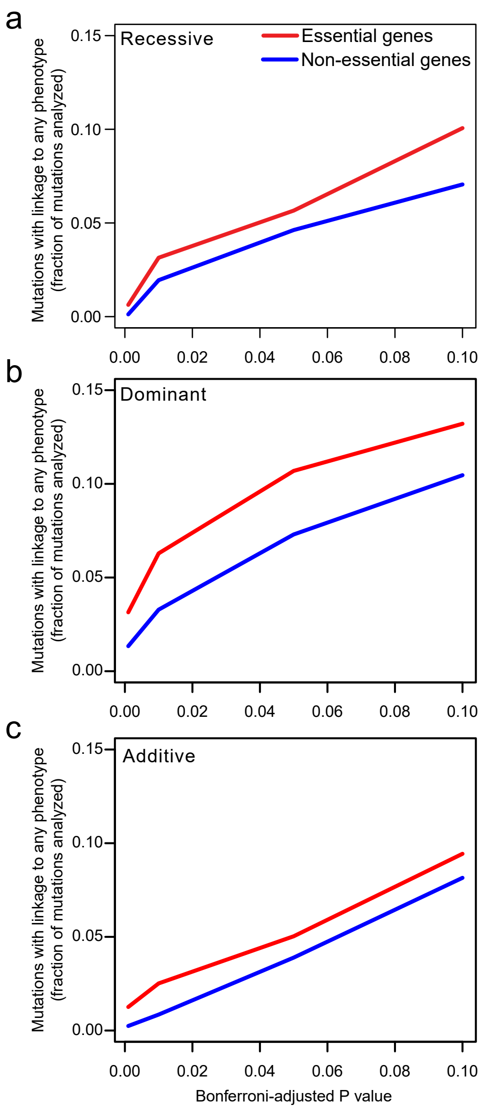

Supplementary Figure 8. Greater frequency of viable phenotypes from mutations in essential genes than from mutations in non-essential genes. The fraction of mutations in essential (red) or non-essential genes (blue) that showed linkage to any phenotype at the indicated Bonferroni-adjusted P values, using (a) recessive, (b) dominant, or (c) additive transmission models. N = 882 non-essential genes, 159 essential genes. Mutations were non-synonymous coding and potential splicing changes of all genes.

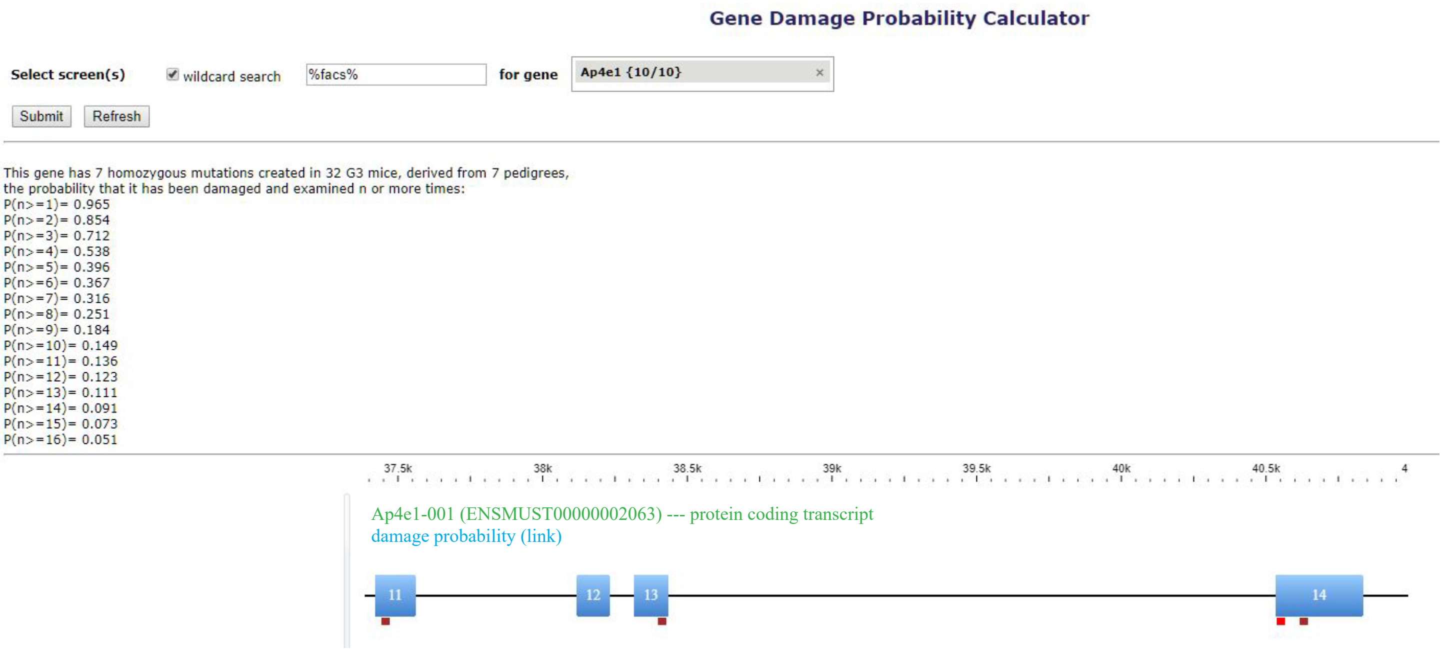

Supplementary Figure 9. Damage probability calculation tool for individual genes. The tool calculates the probability that a gene has been tested for function in at least a specified number of homozygous G3 mice in a particular phenotype screen (among those under surveillance in the lab). The web-based tool is available at https://mutagenetix.utsouthwestern.edu/report/gene_damage/damage_prob.cfm. After submitting the gene and screen(s) in the query form, the program displays two types of results: 1) the number of mutations and mice carrying them used for the probability calculation, along with the list of calculated damage probabilities for specified minimum numbers of G3 mice. 2) Diagrams of all known transcripts: these models can be magnified using the slider on the left. Exons (light blue); 5' and 3' UTRs (dark blue). Mutations are shown as color-coded squares below transcript (green, probably benign; orange, possibly damaging; maroon, probably damaging; red, probably null). Mousing over each mutation mark will display its detailed information. Clicking "damage probability" link will open a new window to summarize the damage probabilities for the designated transcript.

Supplementary Table 1. Lethal annotations reported by MGI.

MGI LETHALITY MP TERM	MGI MP ID	ANNOTATIONS
Embryonic lethality	MP:0008762	408
Embryonic lethality at implantation	MP:0008527	29
Embryonic lethality before implantation	MP:0006204	153
Embryonic lethality between implantation and placentation	MP:0009850	53
Embryonic lethality between implantation and somite formation	MP:0006205	269
Embryonic lethality between somite formation and embryo turning	MP:0006206	88
Embryonic lethality during organogenesis	MP:0006207	748
Embryonic lethality prior to organogenesis	MP:0013292	125
Embryonic lethality prior to tooth bud stage	MP:0013293	161
Lethality*	---	64
Lethality at weaning	MP:0008569	89
Lethality during fetal growth through weaning	MP:0010832	19
Lethality throughout fetal growth and development	MP:0006208	378
Neonatal lethality	MP:0002058	761
Perinatal lethality	MP:0002081	483
Postnatal lethality	MP:0002082	1078
Prenatal lethality	MP:0002080	601
Prenatal lethality prior to heart atrial septation	MP:0013294	51
Preweaning lethality	MP:0010770	1160
**Total**		**6718**

A single gene may have more than one lethality annotation.

Abbreviations: MGI: Mouse Genome Informatics; MP ID: mammalian phenotype identification number.

*Category does not have an associated mammalian phenotype ID on MGI 6.06 (accessed October 2016).

Supplementary Table 2. Estimated damage probabilities for mutations in six PP2 score ranges.

PP2 score range	Mutations	Damage probability	95% CI
0-0.01	205	0.039	0-0.146
0.01-0.4	258	0.058	0-0.142
0.4-0.9	231	0.103	0.012-0.197
0.9-0.99	181	0.111	0.006-0.216
0.99-0.999	189	0.119	0.012-0.227
0.999-1	389	0.199	0.126-0.271

Supplementary Table 3. PP2 and SIFT classifications of missense mutations.

		PP2 classifications		
		Benign	Possibly damaging	Probably damaging
**SIFT classifications**	Deleterious	3513 (11.6%)*	5350 (17.6%)*	21474 (70.8%)*
	Deleterious (Low confidence)	681 (18.7%)*	892 (24.4%)*	2074 (56.9%)*
	Tolerated	20925 (57.4%)*	7639 (21.0%)*	7856 (21.6%)*

*Within each SIFT classification, percentages that were classified by PP2 as benign, possibly damaging, or probably damaging.

Supplementary Table 4. Estimated damage probabilities for mutations classified by several mutation effect prediction algorithms

Classification	Mutations*	Damage probability	95% CI
**PP2 (HumVar)**			
Benign	481	0.05	0-0.115
Possibly damaging	187	0.165	0.062-0.267
Probably damaging	343	0.226	0.146-0.305
**PP2 (HumDiv)**			
Benign	367	0.047	0-0.123
Possibly damging	200	0.133	0.033-0.229
Probably damaging	444	0.199	0.129-0.268
**LRT**			
Unknown	51	0.076	0-0.272
Neutral	323	0.06	0-0.138
Deleterious	614	0.199	0.14-0.257
**MutationAssessor**			
Neutral	165	0.024	0-0.142
Low	342	0.068	0-0.148
Medium	415	0.185	0.115-0.251
High	73	0.423	0.209-0.607
**FATHMM**			
Tolerated	722	0.106	0.05-0.158
Damaging	236	0.2	0.104-0.292
**PROVEAN**			
Neutral	501	0.057	0-0.121
Damaging	488	0.215	0.15-0.278
**MetaSVM**			
Tolerated	753	0.069	0.015-0.122
Damaging	258	0.315	0.228-0.4
**MetaLR**			
Tolerated	738	0.08	0.025-0.134
Damaging	273	0.271	0.187-0.353
**M-CAP**			
Tolerated	372	0.065	0-0.138
Damaging	632	0.169	0.111-0.227
**fathmm-MKL_coding**			
Neutral	149	0.073	0-0.176
Damaging	900	0.157	0.107-0.206

*All mutations represent mouse mutations transferred to the human gene sequences. Only mutations that conferred the identical nucleotide and amino acid change in the mouse and human genes were analyzed.

Supplementary Table 5. Designations of genes as essential or non-essential by MGI and IMPC.

		MGI designations		
		Lethal	Nonlethal	Concordance (%)*
**IMPC designations**	Lethal	635	15	97.7%
	Subviable	250	8	96.9%
	Viable	98	1685	94.5%

*Percentage of genes classified similarly by MGI and IMPC.
