# Supplementary material for: Probability of phenotypically detectable protein damage by ENU-induced mutations in the Mutagenetix database
Source: Nat Commun. 2018 Jan 30;9:441. doi: 10.1038/s41467-017-02806-4 (PMC5789985; doi:10.1038/s41467-017-02806-4)
Supplement: Supplementary file 2 — Description of Additional Supplementary Files [file 41467_2017_2806_MOESM2_ESM.pdf]

Description of Additional Supplementary Files

File Name: Supplementary Data 1
Description: Predicted effects of putative null mutations in 38 non-essential genes.
